# Genomics of sexual cell fate transdifferentiation in the mouse gonad

**DOI:** 10.1093/g3journal/jkac267

**Published:** 2022-10-06

**Authors:** Mark W Murphy, Micah D Gearhart, Andrew Wheeler, Vivian J Bardwell, David Zarkower

**Affiliations:** Developmental Biology Center and Department of Genetics, Cell Biology, and Development, University of Minnesota, Minneapolis, MN 55455, USA; Developmental Biology Center and Department of Genetics, Cell Biology, and Development, University of Minnesota, Minneapolis, MN 55455, USA; Developmental Biology Center and Department of Genetics, Cell Biology, and Development, University of Minnesota, Minneapolis, MN 55455, USA; Developmental Biology Center and Department of Genetics, Cell Biology, and Development, University of Minnesota, Minneapolis, MN 55455, USA; University of Minnesota Masonic Cancer Center, Minneapolis, MN 55455, USA; Developmental Biology Center and Department of Genetics, Cell Biology, and Development, University of Minnesota, Minneapolis, MN 55455, USA; University of Minnesota Masonic Cancer Center, Minneapolis, MN 55455, USA

**Keywords:** DMRT1, transdifferentiation, testis, sex determination, cell fate

## Abstract

Sex determination in mammals hinges on a cell fate decision in the fetal bipotential gonad between formation of male Sertoli cells or female granulosa cells. While this decision normally is permanent, loss of key cell fate regulators such as the transcription factors *Dmrt1* and *Foxl2* can cause postnatal transdifferentiation from Sertoli to granulosa-like (*Dmrt1*) or vice versa (*Foxl2*). Here, we examine the mechanism of male-to-female transdifferentiation in mice carrying either a null mutation of *Dmrt1* or a point mutation, R111G, that alters the DNA-binding motif and causes human XY gonadal dysgenesis and sex reversal. We first define genes misexpressed during transdifferentiation and then show that female transcriptional regulators driving transdifferentiation in the mutant XY gonad (ESR2, LRH1, FOXL2) bind chromatin sites related to those normally bound in the XX ovary. We next define gene expression changes and abnormal chromatin compartments at the onset of transdifferentiation that may help destabilize cell fate and initiate the transdifferentiation process. We model the R111G mutation in mice and show that it causes dominant gonadal dysgenesis, analogous to its human phenotype but less severe. We show that R111G partially feminizes the testicular transcriptome and causes dominant disruption of DMRT1 binding specificity in vivo. These data help illuminate how transdifferentiation occurs when sexual cell fate maintenance is disrupted and identify chromatin sites and transcripts that may play key roles in the transdifferentiation process.

## Introduction

In mammals, sex is determined in the undifferentiated, or bipotential, gonad during fetal development based on their sex chromosome composition (Jost 1947; Albrecht and Eicher 2001). The direct output of sex determination is the differentiation of bipotential precursor cells into either Sertoli cells in males or granulosa cells in females (Lin and Capel 2015). This pivotal cell fate decision is then propagated by signaling pathways that conscript sexual development into the male or the female mode elsewhere in the body. Sex chromosome composition also has direct effects on sexual differentiation in some nongonadal tissues (Arnold 2020). Nevertheless, the sexual cell fate of bipotential precursor cells in the fetal gonad is central to overall anatomical, physiological and behavioral sex differentiation.

Given the pivotal role that Sertoli/granulosa cell differentiation plays in sexual development and reproduction, it is perhaps surprising that the sexual cell fate of these cells can be highly labile after it is specified. Loss of key transcriptional regulators, including the male promoting genes *Dmrt1* and *Sox8/9* or the female promoting genes *Foxl2* and *Esr1/2* can cause male-to-female or female-to-male sexual transdifferentiation (Couse *et al.* 1999; Uhlenhaut *et al.* 2009; Matson *et al.* 2011; Georg *et al.* 2012; Barrionuevo *et al.* 2016). In the case of *Dmrt1* or *Foxl2*, transdifferentiation can be triggered even in differentiated adult gonadal cells, demonstrating that sexual cell fate must be actively maintained during postnatal life (Uhlenhaut *et al.* 2009; Matson *et al.* 2011). Ectopic *Dmrt1* expression in the postnatal ovary also can reprogram granulosa cells into Sertoli-like cells, indicating that DMRT1 can play an instructive role (Lindeman *et al.* 2015; Lindeman *et al.* 2021). How this fundamental cell fate choice can be genetically toggled even long after it is established is a matter of interest both in sexual development and more broadly in cell fate reprogramming.

*Dmrt1* is a member of a transcription factor family sharing the DM domain, a deeply conserved DNA-binding motif first identified in the invertebrate sex regulators *doublesex* and *male abnormal 3* (*mab-3*) (Erdman and Burtis 1993; Raymond *et al.* 1998). Mutations in *Dmrt1* cause severe gonadal dysgenesis in XY mice (Raymond *et al.* 2000) and deletions of distal chromosome 9p causing hemizygosity of *DMRT1* are associated with complete 46, XY gonadal dysgenesis and male-to-female sex reversal in humans (Zarkower and Murphy 2021). Four de novo point mutations in or adjacent to the DM domain of *DMRT1* have been associated with human 46, XY gonadal dysgenesis, strongly suggesting that *DMRT1* is a critical gene for human as well as mouse testicular development (Murphy *et al.* 2015; Chauhan *et al.* 2017; Fan *et al.* 2017; Buonocore *et al.* 2019). However, these human point mutations have not been modeled in mice to confirm their ability to disrupt DMRT1 function and cause gonadal dysgenesis.

DMRT1 can bind DNA as a dimer, trimer or tetramer and, uniquely among transcriptional regulators, DM domain DNA binding involves the coinsertion of 2 antiparallel alpha helices into a widened region of the DNA major groove as well as insertion into an adjacent widened region of the minor groove (Murphy *et al.* 2015). We found previously that reprogramming by DMRT1 involves binding to sex-biased differentially accessible regions (DARs) that are presumed to include sexual cell fate regulatory elements (Lindeman *et al.* 2021). DMRT1 binding to inaccessible male-biased DARs in granulosa cells can render these regions accessible and can facilitate access by other transcriptional regulators such as SOX9, which functionally cooperates with DMRT1 in controlling sexual cell fate (Lindeman *et al.* 2021). Thus, DMRT1 appears to act as a pioneer transcription factor in reprogramming sexual cell fate, imposing Sertoli-like patterns of chromatin accessibility and gene expression on granulosa cells.

Here, we take a genomic approach to examine male-to-female sexual transdifferentiation triggered by loss of DMRT1 in Sertoli cells. Loss of DMRT1 causes ectopic expression of female-promoting transcriptional regulators normally active in the ovary, and genetic analysis has shown that these regulators help drive transdifferentiation (Matson *et al.* 2011; Minkina *et al.* 2014; Agrimson *et al.* 2022). We ask where these transcription factors bind in transdifferentiating Sertoli cells: do they bind with profiles similar to those normally found in granulosa cells or at novel sites; and do they bind jointly or individually to putative sex regulatory elements? We also use HiC to ask how and where loss of DMRT1 affects chromatin conformation at the onset of transdifferentiation. Finally, we create and analyze a mouse model of a point mutation in *DMRT1*, R111G, which is associated with dominant 46,XY complete gonadal dysgenesis in humans and can cause a dominant alteration of DMRT1 DNA binding stoichiometry in vitro (Murphy *et al.* 2015). We show that a *Dmrt1^R111G^* allele in the mouse also causes dominant defects in testicular differentiation and we examine how it affects mRNA expression, hormone levels, and DMRT1 chromatin association, asking whether the phenotype includes gonadal feminization.

## Materials and methods

### Mice and genotyping

Mice were of mixed genetic background (129Sv and C57Bl/6J) and maintained in conventional housing facilities. Presence of a copulation plug in the morning was recorded as day E0.5. Experimental protocols and euthanasia protocols were approved by the University of Minnesota Animal Care and Use Committee (protocol number: 2106-39153A). Genotyping of the wild-type and floxed *Dmrt1* alleles (Raymond *et al.* 2000; Kim *et al.* 2007) used primers CR99 (5′-TGCACACGTGCACCCTCGCCATCG-3′) and CR100 (5′-TCATGGCAGCTCTCCCAGTGGAGC-3′). The deleted *Dmrt1* allele was genotyped with primers CR98 (5′-GATCTATCTGGAGCCAGGTGGTAG-3′) and CR100. The *Dmrt1^R111G^* allele was genotyped using primers R111G-F 5′-GCAGTCTGATCGCGGAGG-3′ and CR100. PCR was performed with 0.5 M betaine at an annealing temperature of 60°C for 32 cycles with a nonproofreading Taq polymerase.

### Generation of Dmrt1^R111G^ mice

R111G mice were generated at the Cornell University Stem Cell and Transgenic Core Facility using the crRNA + tracrRNA system from Integrated DNA Technologies (IDT; Coralville, IA) as well as a homologous repair DNA template from IDT [essentially as described by Gao *et al.* (2021)]. The CRISPR guide sequence was CCCGCTGTCGCTCCGCAATC and the repair template was 5′-GGCCACAAGCGCTTCTGCATGTGGCGGGATTGCCAGTGCAAGAAGTGCAG**T**CTGAT**C**GCGGA G**G**GACAGCGGGTGATGGCCGCGCAGGTGGCCCTGAGAAGACAGCAGGCCCA-3′. The 2 5′-most bold underlined nucleotides in the repair template are silent changes that remove the PAM sequence and generate a restriction enzyme recognition site, respectively. The third bold underlined nucleotide generates R111G. Two hundred and thirty-two-cell embryos were implanted, resulting in 53 pups, 4 of which had the desired change, based on PCR. Of these, 1 animal was shown to have a correctly edited allele and was bred to establish the mutant line. The *Dmrt1^R111G^* mouse line will be available from The Jackson Laboratory as JAX#037723.

### Sertoli cell isolation

Testes were dissected from P7 mice carrying *CAG-Stop^flox^-tdTomato* driven by *Dhh-Cre.* Testis tubules were manually dissected and loosened with forceps and suspended in 800 µl Dulbecco’s phosphate-buffered saline (DPBS; Gibco 14190-144) with 4 tubule bundles (from 2 mice) per 2 ml microfuge tube. Then, 50 μl Col1A (5 mg/ml; Sigma C5894) and 1 μl DNAse (10 mg/ml, Roche 10104159001) were added and tubules were incubated at 37°C for 10 min with rotation. After incubation, tubules were allowed to settle, then were rinsed 3–6 times with DPBS, centrifuging the tubules for 5 s at 50× g between each subsequent rinse. The rinsed tubule pellet was resuspended in 200 μl trypsin/EDTA with pipetting to break up any remaining clumps and incubated at 37°C for 10 min with rotation, and then an additional 600 μl trypsin/EDTA (Sigma 57428C) was added followed by an additional 10 min incubation at 37°C with rotation. Two hundred microliters fetal bovine serum (Gibco 26140-079) and 50 μl DNAse (10 mg/ml; Worthington LS002139) were added to each tube followed by a 5-min incubation at 37°C with rotation. Cells were then filtered through a 40-μm mesh, pelleted at 1,500 rpm in a clinical centrifuge, transferred to a 2-ml microfuge tube, and resuspended in 300 μl DPBS with 0.5% FBS. Next, 5 μl Thy1 and 5 μl c-Kit antibody beads (Miltenyi Biotec, 130-049-101 and 130-091-224) were added followed by a 15 min incubation at 4°C and the cell mixture was applied to an equilibrated LS separation column (Miltenyi, 130-042-401) on a Miltenyi magnetic stand. Flow-through, enriched for Sertoli cells, was collected along with a 1-ml rinse. Cells were examined by epifluorescence microscopy to determine the number and purity of tdTomato-positive Sertoli cells, and all cell preparations used were at least 90% pure.

### Hormone measurements

Testicular and blood serum testosterone measurements, blood serum luteinizing hormone (LH), blood serum follicle stimulating hormone (FSH), and blood serum inhibin-B for control and mutant mice were performed by The University of Virginia Center for Research in Reproduction Ligand Assay and Analysis Core, which is supported by the Eunice Kennedy Shriver NICHD/NIH Grant R24HD102061. Statistical analyses of hormone measurements were performed using the Mann–Whitney *U* test excluding samples that were above or below the limits of detection.

### Histology and immunofluorescent staining

Histology and immunofluorescence (IF) was performed on 5 μm sections of paraffin-embedded tissue previously fixed in 4% paraformaldehyde (Electron Microscope Sciences 15710) diluted in PBS. Single animals were used for each timepoint in each method. Antigen retrieval for IF was accomplished by boiling in 10 mM citrate (pH 6.0) for 1 h. Prior to blocking, cells were permeabilized in 1× DPBS with 0.1% Triton X-100 at room temperature for 10 min. After blocking with donkey serum (Sigma-Aldrich S30-100ML) diluted to 10% in 1× DPBS with 0.1% BSA (Sigma A9647) and 0.1% Tween 20 (Sigma P9416) at room temperature for 1 h, slides were stained overnight with rat anti-TRA98 (1:200) (Abcam ab82527) and rabbit anti-SOX9 (1:200) (EMD Millipore AB5535) at 4°C. Secondary antibodies used were Alexa Fluor 488 donkey anti-rat IgG (1:500) (Invitrogen A21208) and Alexa Fluor 594 donkey anti-rabbit IgG (1:500) (Invitrogen A21207). Slides were counterstained with 1 µg/ml DAPI (Thermo Scientific 62248) in DPBS to detect nuclei. Slides were sealed with cover slips (Fisher Scientific 22037298) in mounting medium (Permafluor, Thermo Scientific TA030FM).

### Image capture

IF images were captured with a Zeiss Axio Imager Z1 microscope and Zeiss MRm camera, processed and false-colored using Zeiss Axiovision software. High magnification images were captured using Zeiss Apotome structured illumination. Histology (hematoxylin and eosin) images were captured with a Leica DMRB microscope using a Zeiss MRc camera and processed using Zeiss MRGrab software.

### BTB analysis

Biotin permeability was assayed essentially as described (Pérez *et al.* 2012; Jauregui *et al.* 2018) using freshly dissected testes from 3.5-, 16-, and 20-week-old animals. Ten milligrams per milliliters of EZ-Link Sulfo-NHS-LC-Biotin (Pierce 21335) diluted in 1× PBS containing 1 mM CaCl_2_ was injected into 1 testis while the other was injected with vehicle. Testis sections were stained with Alexa Fluor 594 conjugated streptavidin (Invitrogen S32356).

### mRNA sequencing

Testes were harvested and homogenized in Trizol reagent (Thermo Fisher) and stored at −80°C prior to processing. Total RNA was extracted from the aqueous phase, mixed with ethanol and purified using the RNeasy kit protocols and reagents (Qiagen). RNA was quantified using the Qubit RNA assay (Thermo Fisher) and 400–500 ng total RNA per sample was used in stranded mRNA-seq library preparation (KAPA Biosystems, KK8481) for Illumina sequencing. Libraries were pooled and sequenced with 2×150 cycles paired-end to an average depth of 19.4 million reads per sample on a HiSeq 4000 by Genewiz (South Plainfield, NJ, USA).

### ChIP-seq

For ChIP-seq, freshly isolated Sertoli cells were fixed with 1% methanol-free formaldehyde in PBS (Thermo scientific #28906) for 5 min at RT with rotation. Fixation was stopped by addition of glycine to 0.125 M and rotation at RT for 10 min. After pelleting, cells were washed in PBS and stored at −80°C until use. ChIP was performed with 1–2.5 million cells. Nuclei were prepared from the fixed cells and chromatin was sheared to an average size of 300–400 bp in a Covaris S220 according to the manufacturer’s recommendations. After shearing, the lysate was diluted 1:3 with complete DOC RIPA (Murphy *et al.* 2010) and the sample was centrifuged at 21,000 g for 10 min at 4°C to pellet any insoluble material. The supernatant was transferred to a fresh siliconized tube and the ChIP was initiated with the addition of ∼1 µg of the relevant antibody and rotated at 4°C overnight. Samples were then spun at 21,000 g for 10 min to pellet insoluble material and supernatant was mixed with 20 µl Protein A Dynabeads (Invitrogen, 10002D) previously blocked with BSA and yeast tRNA to inhibit nonspecific binding. After incubation at 4°C for 60 min with rotation to allow association of protein A with the antibody, beads were applied to a magnet and washed sequentially as described previously (Murphy *et al.* 2010). After elution and cross link reversal, sequencing libraries were prepared using the Hyper Prep Kit (KAPA Biosystems, KK8502).

Whole gonad ChIP-seq was performed essentially the same as for primary cells ChIP-seq except gonads from 2 to 3 P28 animals were pooled and fixed in 1% paraformaldehyde (Electron Microscopy Sciences #15710) for 10 min. After quenching the fix with 0.125 M glycine, chromatin was sheared to an average size of 300–500 bp using a tip sonicator. Antibodies used were anti-ESR2 (Aviva Biosystems ARP37039), anti-LRH1 (Aviva Biosystems ARP37408), and anti-FOXL2 (Aviva Biosystems ARP39574).

### Hi-C

Two independent replicates of Hi-C were performed for each genotype on about 1 million primary Sertoli cells using the Arima Hi-C Kit (#A510008). Cells were fixed in 2% methanol-free formaldehyde in DPBS at RT for 10 min with occasional mixing then quenched by addition of glycine to 0.125 M with occasional mixing for 5 min. Cells were placed on ice for 15 min prior to washing with 1× DPBS and stored at −80°C. Hi-C was performed according to Arima Genomics (Document part # A160134 v00). Sequencing libraries were prepared using the KAPA Hyper Prep Kit and xGen Duplex Seq adapters (Integrated DNA Techologies) according to Arima Genomics (Document part # A160139 v00).

### Bioinformatic analysis

High-throughput Illumina sequencing was performed by the University of Minnesota Genomics Center or GENEWIZ (South Plainfield, NJ, USA) using a combination of HiSeq 2500, HiSeq 4000, and NovaSeq platforms. For each of the experiments, reads were trimmed using Trim Galore (v0.6.0) and cutadapt (v1.18) and assessed for quality with FastQC (v0.11.8). STAR (v2.7.2a), BWA mem (v0.7.12), and HiC-Pro (v3.11.0) were used to map transcriptomic (RNA-seq), genomic (ChIP-seq) and 3D (Hi-C) trimmed reads to the GRCm38 (mm10) genome, respectively. The GENCODE M25 gene annotation set was used to estimate strand-specific gene expression data in the RNA-seq data with the Bioconductor package RSubread (v1.28.1) and to assign the locations of ChIP peaks to nearby genes. For ChIP-seq experiments, duplicated reads were removed with Picard MarkDuplicates (v2.17.10) and low-quality reads (MAPQ < 55) were removed with SAMtools (v1.0). Hi-C data were processed using the LIGATION_SITE = GATCGATC, GATCGANTC, GANTCGATC, GANTCGANTC, and GET_PROCESS_SAM = 1 options within HiC-Pro. Contact matrices were normalized and converted to cool format with cooler (v0.8.11) and visualized with HiGlass (v1.9.4). Statistically enriched contact interactions and A/B compartments were identified with cooltools (v0.5.1). MACS2 (v2.1.1.20160309) was used to call peaks in the ChIP-seq datasets using the parameters “-call-summits -m 2 20.” Enriched DNA-binding motifs were identified using MEME (v5.0.1) using the parameters “-maxsize 900000000 -searchsize 0 -text -dna -revcomp -nmotifs 3 -p 1 -nostatus.” Analysis of predicted minor groove widths was performed using DNAshapeR (v1.24.0) (Chiu *et al.* 2016).

## Results

### Transcriptome feminization in *Dmrt1* mutant testes

**Fig. 1.**
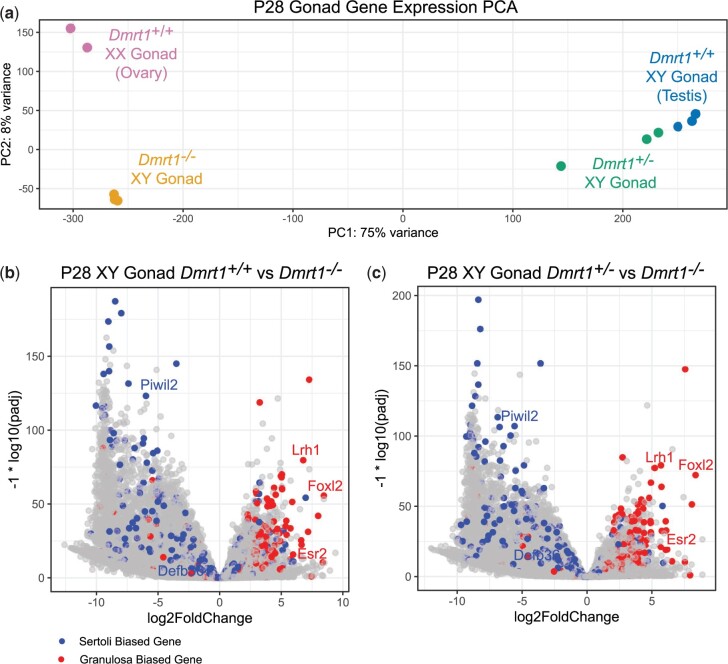
Transcriptome feminization in *Dmrt1* mutant XY gonads. mRNA-seq data from P28 wild-type and *Dmrt1^+/−^* XY testes, P28 *Dmrt1^−/−^* XY gonads and 2- to 3-month wild-type XX ovaries. a) Plot of PCA analysis showing clustering of wild-type with *Dmrt1^+/−^* testes and of *Dmrt1^−/−^* XY gonads with wild-type ovaries in PC1. b) Volcano plot comparing mRNA-seq data from wild-type testes and *Dmrt1^−/−^* XY gonads showing differentially expressed genes, with previously identified Sertoli-biased transcripts in blue and granulosa-biased transcripts in red. c) Volcano plot comparing *Dmrt1^+/−^* testes with *Dmrt1^−/−^* XY gonads, showing very similar pattern to comparison in panel B.

We showed previously that deletion of *Dmrt1* in the testis causes a major transformation of the transcriptome toward a more ovary-like mRNA profile (Matson *et al.* 2011). Because the earlier study employed microarray analysis, we first used mRNA-seq to gain a more complete comparison of transcriptomes from wild-type testes and ovaries relative to XY gonads homozygous or heterozygous for a *Dmrt1* null mutation. We examined gonads at P28, when transdifferentiation is advanced in mutant XY gonads (Matson *et al.* 2011). As expected, in principal component analysis (PCA) wild-type testis and ovary profiles were widely separated in principal component 1 (PC1), which accounted for 75% of variance (Fig. 1a). Heterozygous XY gonads, which have no overt phenotype (Raymond *et al.* 2000; Kim *et al.* 2007), clustered close to wild-type testes. Homozygous *Dmrt1* mutant XY gonads clustered with wild-type ovaries in PC1, consistent with our previous studies showing their extensive feminization (Matson *et al.* 2011; Minkina *et al.* 2014). PC2 accounted for 8% of variance. As might be predicted, a number of the top differentially expressed genes driving PC2 were X- or Y-linked genes or genes involved in folliculogenesis.

To examine the molecular phenotype in more detail, we generated volcano plots of differentially expressed mRNAs, with known Sertoli- and granulosa-biased mRNAs (Lindeman *et al.* 2021) indicated in blue and red, respectively (Fig. 1b and c). As expected from PCA and previous phenotypic analysis, comparison of homozygous mutant XY gonads vs wild-type testes revealed mainly downregulation of Sertoli-biased mRNAs and upregulation of granulosa-biased mRNAs (Fig. 1b). We identified 9,151 downregulated and 4,882 upregulated mRNAs with a >2-fold change and a Benjamini–Hochberg adjusted *P*-value below 0.05 (Supplementary Table 1). Among the strongly upregulated granulosa-biased mRNAs are *Foxl2*, *Esr2*, and *Nr5a2/Lrh1*, which encode transcription factors important in ovarian development and sexual transdifferentiation of *Dmrt1* mutant XY gonads (Minkina *et al.* 2014; Agrimson *et al.* 2022). Importantly, comparison of *Dmrt1* null XY gonads with those of *Dmrt1^+/−^* heterozygotes gave virtually identical results, suggesting that the *Dmrt1* null allele is highly recessive at the molecular as well as the phenotypic level (Fig. 1c;Supplementary Table 1).

### Loss of DMRT1 activates chromatin binding by feminizing transcription factors

We next examined the role of FOXL2, ESR2, and LRH1 in sexual transdifferentiation by asking where they bind chromatin in wild-type young adult (2–3 month) ovaries vs P28 mutant XY gonads undergoing transdifferentiation. Genetic analysis has established that all 3 regulators are important drivers of transdifferentiation of *Dmrt1* mutant Sertoli cells into granulosa-like cells (Minkina *et al.* 2014; Agrimson *et al.* 2022) but the mechanisms underlying this cell fate reprogramming have not been investigated. We sought to address 2 key questions: first, do these regulators bind similar regions in ovaries and transdifferentiating mutant gonads; and second, do they bind to distinct sites or might they cooperatively control shared regulatory elements? We used ChIP-seq to compare binding by each protein in wild-type ovaries, *Dmrt1* heterozygous mutant testes, and *Dmrt1* homozygous mutant XY gonads undergoing transdifferentiation.

**Fig. 2.**
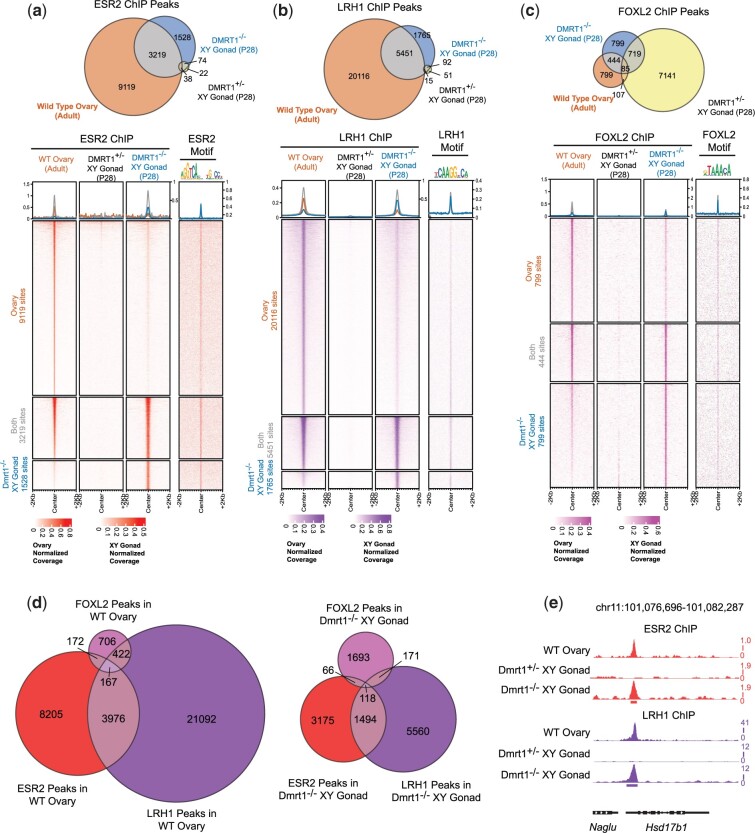
Overlapping DNA binding by feminizing transcription factors in *Dmrt1* mutant XY gonads and XX ovaries. a–c) Comparison of DNA binding by ESR2, LRH1, and FOXL2 in wild-type young adult ovaries, *Dmrt1^+/−^* XY P28 testes, and *Dmrt1^−/−^* XY P28 gonads. Venn diagrams indicate the extent of overlap in binding and tornado plots show intensity of binding at sites bound primarily in ovaries, primarily in homozygous mutant XY gonads, or in both. Matches to consensus DNA-binding motif for each protein are indicated in far right columns. d) Venn diagram showing extent of overlapping binding by the three feminizing transcription factors in *Dmrt1^−/−^* XY gonads.

We first used ChIP-seq to examine binding by the estrogen receptor ESR2, a nuclear hormone receptor transcription factor normally expressed and active in granulosa cells (Lenie and Smitz 2008). ESR2 bound many sites in ovaries and in homozygous mutant gonads, with more than twice as many sites bound in ovary (12,338 vs 4,747) (Fig. 2a). In contrast, only 134 bound sites were detected in heterozygous testes, consistent with the very low *Esr2* expression in these gonads (Matson *et al.* 2011). A majority of sites bound strongly in the homozygous mutant gonad (3,219/4,747) also were bound strongly in the ovary, and most sites strongly detected in 1 class of gonad also were detected in the other, though often below a false discovery q-value cutoff of 0.05 for peak calling. Sites bound in the homozygous mutant XY gonad, the ovary, or both, were enriched for the ESR2 consensus DNA-binding motif (Fig. 2a, far right column), suggesting good ChIP specificity. These data indicate similar binding by ESR2 in the ovary and mutant XY gonad and strongly suggest that estrogen signaling is active in chromosomally male gonads when *Dmrt1* is missing.

Next, we examined binding by the orphan nuclear receptor LRH1 (NR5A2). LRH1 is the closest relative of the steroidogenic regulator SF1 and plays an essential role in granulosa cell differentiation and function (Duggavathi *et al.* 2008; Meinsohn *et al.* 2018; Meinsohn *et al.* 2021). Similar to ESR2, ChIP-seq showed that LRH1 bound many sites in ovaries and in homozygous mutant XY gonads, with more sites bound in ovaries (25,567 vs 7,216), while only 158 bound sites were detected in heterozygous testes (Fig. 2b). As with ESR2, a majority of sites strongly bound by LRH1 in mutant XY gonads (5,451/7,216) also were strongly bound in the ovary and sites bound in the mutant XY gonad, the ovary, or both, were enriched for the LRH1 consensus DNA-binding motif (Fig. 2b, far right column). Also similar to ESR2, most sites bound strongly in ovaries or mutant testes also were detected in the other, although often not called as a peak using a *q*-value cutoff of 0.05. These data indicate that LRH1, like ESR2, binds a similar repertoire of sites in the wild-type XX ovary and the transdifferentiating *Dmrt1* mutant XY gonad.

Finally, we examined binding by the forkhead transcription factor FOXL2, which is required for normal granulosa cell differentiation and for maintenance of female differentiation in the ovary (Uda *et al.* 2004; Uhlenhaut *et al.* 2009). Like ESR2 and LRH1, FOXL2 is ectopically expressed in *Dmrt1* mutant XY gonads and is essential for transdifferentiation of mutant Sertoli cells to granulosa-like cells (Matson *et al.* 2011; Minkina *et al.* 2014). Once again, we found that FOXL2 binds many sites in ovaries and in homozygous mutant XY gonads, with a similar number of sites bound in each (1,467 in ovary vs 2,048 in mutant XY gonad) (Fig. 2c). As with the other proteins, most sites strongly bound by FOXL2 in ovaries or mutant gonads were bound at least weakly in the other. An unexpected result was that many bound sites (8,052) were detected in *Dmrt1* heterozygous testes, which express extremely low levels of FOXL2 (Fig. 2c). We considered 2 possibilities. First, the antibody might not recognize FOXL2. This appears not to be the case, as the sites we detected in ovaries were highly enriched for the canonical FOXL2-binding consensus (Fig. 2c, far right column) and showed strong overlap with sites detected in cultured follicles using a different FOXL2 antibody (Supplementary Fig. 1) (Georges *et al.* 2014). Binding was weaker in intact ovaries than cultured cells, either because this antibody has lower affinity or because the cells expressing FOXL2 are a minority of cells present in the intact ovary, resulting in a lower signal to noise ratio. However, it appears that both antibodies detect binding to the same suite of DNA elements in ovarian cells. Second, we considered that the antibody may not only bind FOXL2 but also cross-react with another protein expressed in testes. This possibility appears likely, as there was low overlap between sites bound in wild-type testes and those bound in ovaries or cultured granulosa cells (Fig. 2c;Supplementary Fig. 1). Also, the sites bound strongly in testes and weakly in ovaries or mutant XY gonads were not enriched for the FOXL2-binding motif and instead were enriched for a string of A/T basepairs (Supplementary Fig. 2). We therefore conclude that FOXL2 has similar binding in ovaries and transdifferentiating mutant XY gonads, but that the antibody likely also crossreacts with a second unidentified protein that is more detectable in *Dmrt1* heterozygous testes in the absence of FOXL2 expression.

### Feminizing transcription factors bind common regulatory elements

ESR2, LRH1, and FOXL2 all help drive transdifferentiation in *Dmrt1* mutant XY gonads (Minkina *et al.* 2014; Agrimson *et al.* 2022). A key question is whether they do so by binding to distinct or overlapping sets of regulatory elements. Both ESR2 and LRH1 recognize the sequence AGGTCA in the major groove, although ESR2 binds as an inverted dimer while LRH1 binds as a monomer to an extended half-site (Schwabe *et al.* 1993; Fayard *et al.* 2003). We found that ESR2 and LRH1 have substantially overlapping binding in P28 *Dmrt1^−/−^* XY gonads, with 1,612 bound by both proteins, vs 3,241 bound only by ESR2 and 5,731 bound only by LRH1 (Fig. 2d;Supplementary Table 2). Thus, about one-third of ESR2 sites also are bound by LRH1 and about one-fifth of LRH1 sites also are bound by ESR2. An example of binding by ESR2 and LRH1 at the sex steroid regulator *Hsd17b1* is shown in Fig. 2e. FOXL2 showed less binding overlap with ESR2 and LRH1. Of 2,047 sites bound by FOXL2 in the mutant XY gonad, only 184 also were bound by ESR2 and 289 were bound by LRH1. We conclude that ESR2 and LRH1 bind to an overlapping set of regulatory elements and may cooperate in some manner to regulate target genes, while FOXL2 appears to bind a mostly distinct set of elements.

### DMRT1 regulates sex-biased mRNA expression in neonatal Sertoli cells

**Fig. 3.**
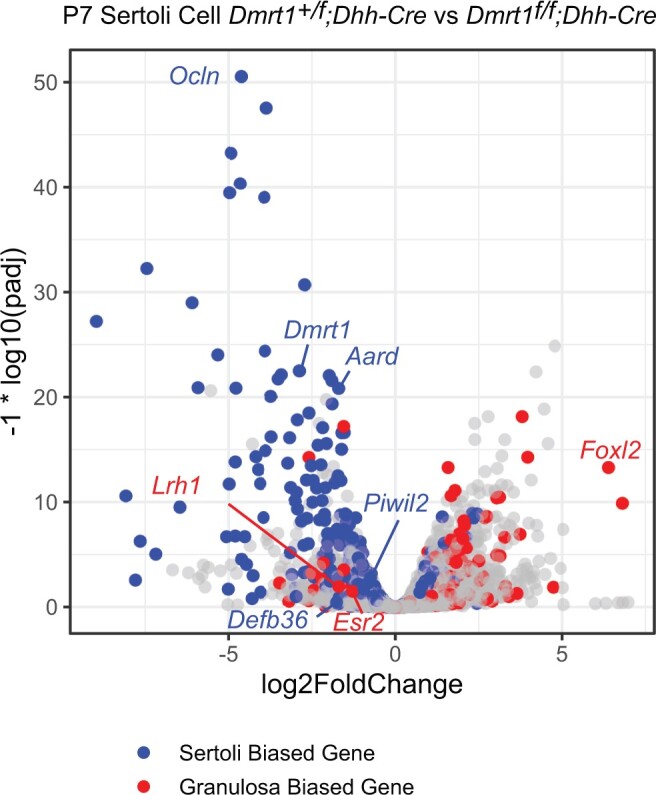
Gene expression changes in P7 *Dmrt1* mutant Sertoli cells. Volcano plot showing mRNAs misexpressed in *Dmrt1* mutant P7 Sertoli cells. mRNAs with sex-biased expression from Lindeman *et al.* (2021) are colored blue (Sertoli-biased) or red (granulosa-biased).

Previous analyses of transdifferentiation triggered by *Dmrt1* loss have analyzed adult gonads or relied on limited protein markers (Matson *et al.* 2011; Minkina *et al.* 2014). To better understand the onset of the process we therefore examined isolated juvenile Sertoli cells. We first examined gene expression by RNA-seq comparison of *Dmrt1* heterozygous and homozygous mutant isolated P7 Sertoli cells. We identified 426 down regulated genes and 512 upregulated genes with a greater than 2-fold change and a Benjamini–Hochberg adjusted *P*-value below 0.05 (Supplementary Table 1). Strikingly, even at this early stage, misexpressed mRNAs included many previously shown to be Sertoli- or granulosa-biased (Fig. 3). Among these, most of the male-biased mRNAs showed reduced expression in mutant cells and most of the female-biased mRNAs showed elevated expression, indicating that sexual transdifferentiation is under way by the end of the first postnatal week. *Esr2* and *Lrh1* were 2 sex regulators that behaved differently at this early stage. These genes are important regulators of granulosa development that promote male-to-female transdifferentiation (Minkina *et al.* 2014; Agrimson *et al.* 2022). Both mRNAs are strongly elevated in P28 *Dmrt1* mutant XY gonads (Fig. 1b) (Matson *et al.* 2011), but both were reduced in P7 mutant Sertoli cells (Fig. 3). This result likely reflects the roles that *Esr2* and *Lrh1* play in differentiation of juvenile Sertoli cells before they become strongly female-biased and regulate granulosa cell differentiation and function (Macheroni *et al.* 2020; Agrimson *et al.* 2022).

### DMRT1 regulates sex-biased chromatin conformation in juvenile Sertoli cells

**Fig. 4.**
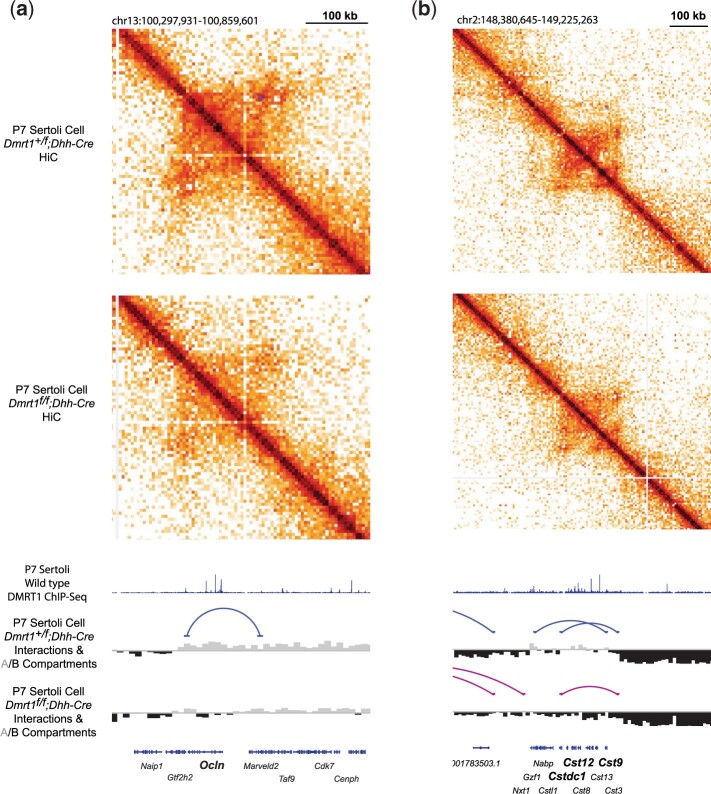
Altered three-dimensional genome organization in P7 *Dmrt1* mutant Sertoli cells. HiC contact maps (top) and one-dimensional tracks for two regions surrounding the male-biased genes *Ocln* (a) and *Cst9*, *Cst12*, and *Cstdc1* (b). Enriched off-diagonal contacts for wild-type or mutant Sertoli cells using 10 kb binning of the contact maps are shown in top panels. One-dimensional representations of the enriched off-diagonal contacts, A/B compartments calculated from the eigenvalues of the interaction matrix, and DMRT1 ChIP-seq data as well as genomic features are shown in the tracks in the bottom panels.

Sex-biased genes are associated with chromatin regions that are found in active “A” compartments or inactive “B” compartments corresponding to the sex in which they are more highly expressed (Garcia-Moreno *et al.* 2019; Lindeman *et al.* 2021). To identify possible regulatory foci of DMRT1, we used Hi-C to identify 3D chromatin interactions and asked whether DMRT1 is required to maintain A or B compartments at the onset of transdifferentiation. In isolated P7 Sertoli cells we found DMRT1-dependent transitions between the compartment types (A to B or vice versa) in *Dmrt1* mutant XY gonads for 44 downregulated and 37 upregulated genes, including the male-biased genes *Ocln*, *Cst9*, *Cst12*, and *Cstdc1* (Fig. 4) and the female-biased gene *Zpf521* (Supplementary Fig. 3). These data suggest that DMRT1 likely maintains male sexual cell fate in part by directly or indirectly maintaining appropriate chromatin status, both active and inactive, and they identify a set of potential regulatory elements likely to play a pivotal early role in sex maintenance and transdifferentiation.

### R111G allele of *Dmrt1* has a dominant testis phenotype

In humans, deletions and microdeletions affecting DMRT1 are associated with defects in testicular differentiation including complete gonadal dysgenesis in XY individuals (Zarkower and Murphy 2021). The human *DMRT1^R111G^* point mutation alters a conserved residue in DMRT1 that plays a central role in DNA binding and it is associated with dominant XY complete gonadal dysgenesis, resulting in male-to-female sex reversal (Murphy *et al.* 2015). We showed previously that DMRT1^R111G^ protein can alter the stoichiometry of DNA binding by wild-type DMRT1, potentially helping explain the dominant in vivo phenotype of the mutation (Murphy *et al.* 2015). To further investigate the effects of the R111G mutation, we used the CRISPR/Cas9 system to generate mice carrying this missense mutation in *Dmrt1*.

**Fig. 5.**
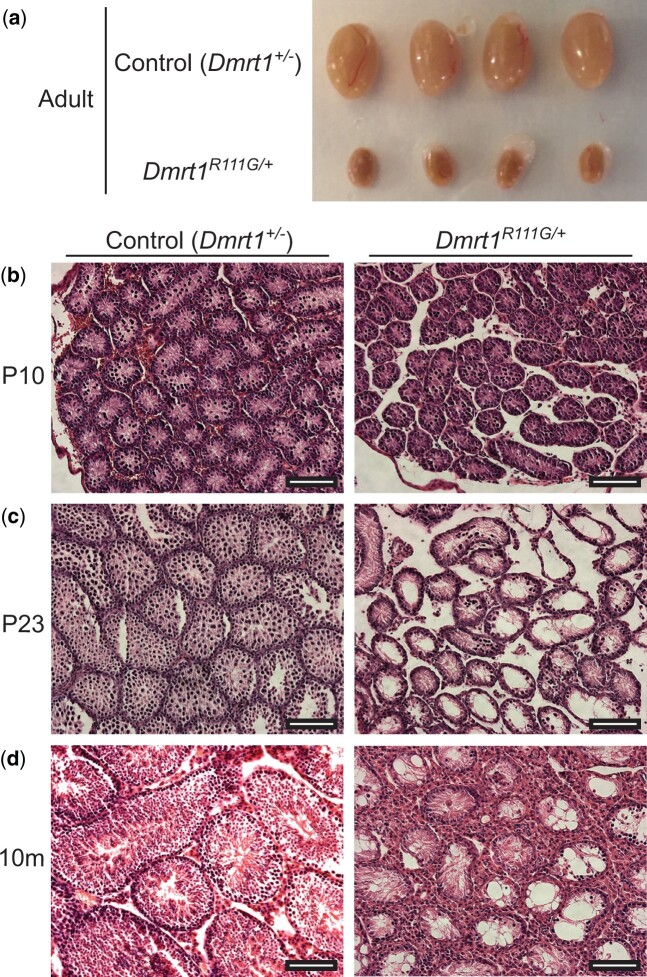
Histology of *Dmrt1^R111G/+^* XY gonads (a) Intact gonads from adult control (*Dmrt1^+/−^*) XY testes and *Dmrt1^R111G/+^* XY testes. (b–d) Hematoxylin/eosin (H&E) staining of sections from control and *Dmrt1^R111G/+^* XY testes at indicated ages. Scale bars: 100 µm.

**Fig. 6.**
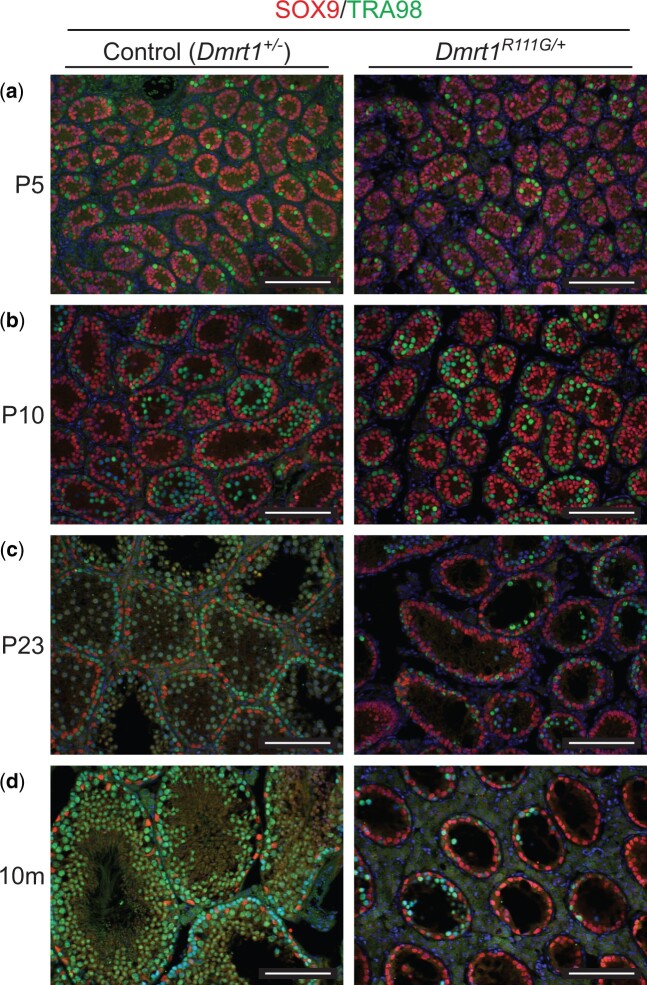
IF analysis of *Dmrt1^R111G/+^* XY gonads. (a–d) IF of sectioned control (*Dmrt1^+/−^*) XY testes and *Dmrt1^R111G/+^* XY testes at indicated ages, stained for Sertoli marker SOX9 (red) and germ cell marker TRA98 (green). Scale bars: 100 µm.

We found that *Dmrt1^R111G^*^/+^ XY mice are phenotypically male but are infertile and show dominant defects in testis differentiation, resulting in hypoplastic adult testes (Fig. 5a). XX heterozygotes are fertile and were used to propagate the strain. Staining of XY gonads with hematoxylin and eosin (H&E) showed that seminiferous tubule size and germ cell abundance appeared normal from birth to P10 but germ cells were severely depleted from P23 onward (Fig. 5b–d). To examine this phenotype more closely we performed IF staining for the Sertoli cell marker SOX9 and the germ cell marker TRA98 (Fig. 6). Consistent with the H&E staining, IF showed apparently normal tubules at P5 and P10 (Fig. 6a and b) but depletion of differentiating germ cells in *Dmrt1^R111G^*^/+^ testes by P23 (Fig. 6c), with only small numbers of spermatogonia present in adult testes (Fig. 6d). These data suggest that *Dmrt1^R111G^*^/+^ testes have a defect in spermatogonial differentiation, meiosis, or both.

**Fig. 7.**
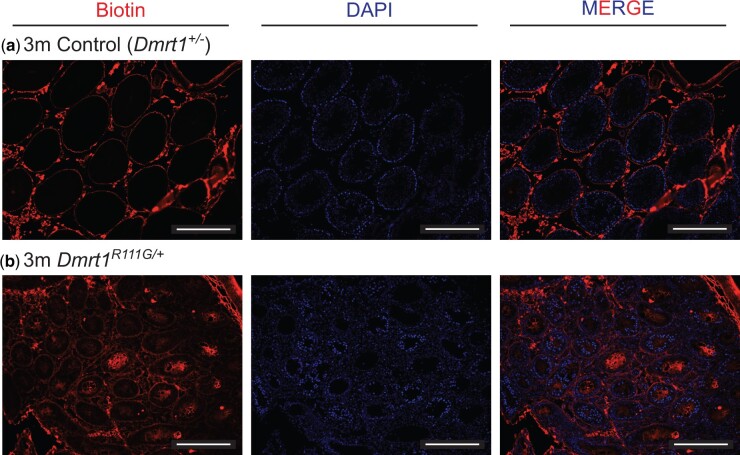
Blood–testis barrier defects in *Dmrt1^R111G/+^* XY gonads. Fluorescence images of sectioned 5-month control (*Dmrt1^+/−^*) XY testis (a) and *Dmrt1^R111G/+^* XY testis (b) injected with biotin and stained with fluorescent streptavidin (red) and DAPI (blue). Scale bars: 100 µm.

Sertoli cells normally form tight junctions during puberty to create the blood–testis barrier (BTB), which is required for the survival of meiotic and postmeiotic germ cells (Kaur *et al.* 2014). Because *Dmrt1^R111G^*^/+^ adult testes have spermatogonia but not more advanced germ cells, we asked whether the BTB was disrupted. We tested BTB integrity by interstitial injection of biotin at 3.5, 16, and 20 weeks. As expected, all tubules in control testes efficiently excluded biotin, but virtually all tubules in the *Dmrt1^R111G^*^/+^ testes were highly biotin-permeable at all ages tested (100% of tubules permeable at 20 weeks, *n* = 50; Fig. 7). From this result we conclude that a defective BTB, due to incomplete Sertoli cell differentiation, is likely to contribute to germ cell deficiency in *Dmrt1^R111G^*^/+^ testes.

### *Dmrt1^R111G^* disrupts the hypothalamic–pituitary–gonadal axis

**Fig. 8.**
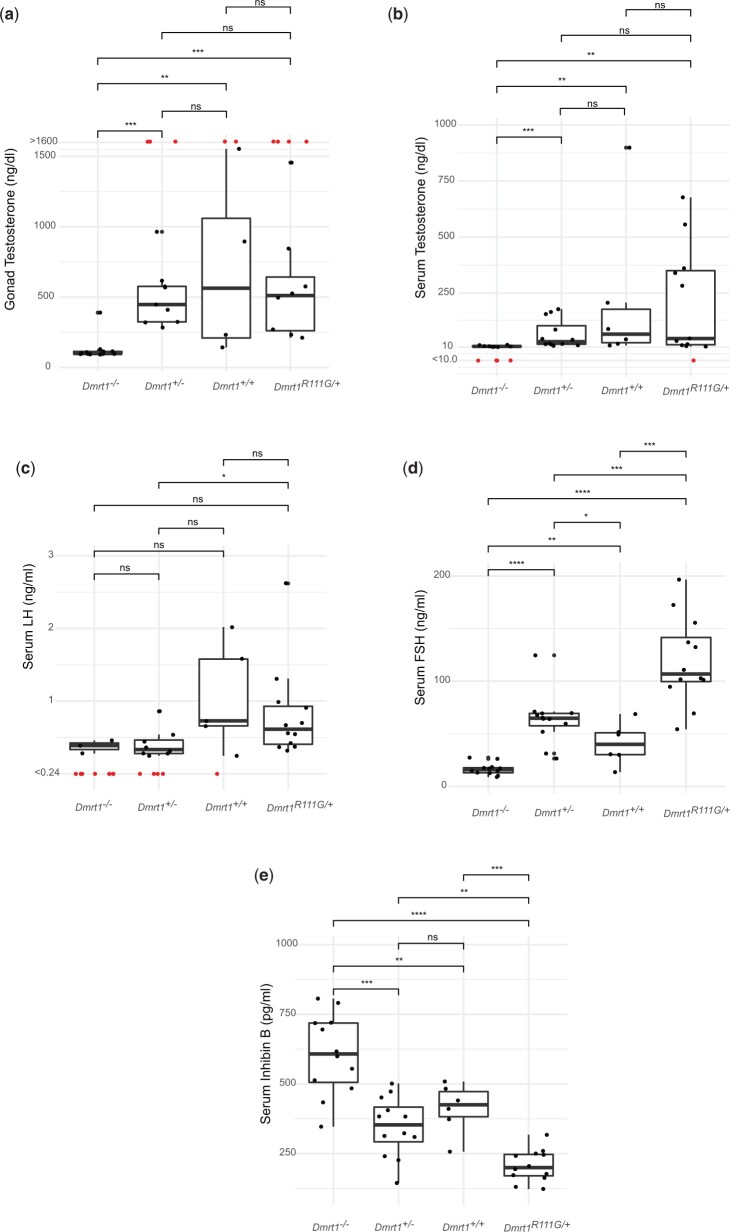
Dmrt1 mutations disrupt the HPG axis. Box plots show individual values, mean, and SD for hormone levels in *Dmrt1^−/−^* (*N* = 12), *Dmrt1^+/−^* (*N* = 12), *Dmrt1^+/+^* (*N* = 6), and *Dmrt1^R111G/+^* (*N* = 12) XY animals. Hormones assayed were gonadal testosterone (a), serum testosterone (b), serum LH (c), serum FSH (d), and serum inhibin B (e). Statistical significance (Mann–Whitney test) is indicated: ns, *P* > 0.05; *, *P* ≤ 0.05; **, *P* ≤ 0.01; ***, *P* ≤ 0.001; ****, *P* ≤ 0.0001. Values above or below the assay threshold are shown in red and were not included in statistical analysis.

To learn more about broader effects of *Dmrt1* mutations, we examined hormone signaling in 6 wild-type adult males and twelve males each that were heterozygous for the null allele, homozygous for the null allele, or heterozygous for R111G (Fig. 8). Sertoli cells respond to FSH from the anterior pituitary and signal back with inhibin. Leydig cells respond to LH from the anterior pituitary and produce testosterone, which signals to Sertoli cells and also back to the pituitary and hypothalamus. Although testes of *Dmrt1^-/+^* XY animals appear phenotypically normal and they are fully fertile males, we did detect minor differences in hypothalamic–pituitary–gonadal (HPG) axis signaling, suggesting that loss of *Dmrt1* may be very weakly haploinsufficient. In particular, while gonadal and serum testosterone levels were not significantly different from wild-type (Fig. 8a and b), serum LH was reduced and serum FSH was slightly elevated (Fig. 8c and d), suggesting an effect on HPG axis signaling. Serum inhibin B was normal, however, suggesting functional Sertoli cell feedback (Fig. 8e). In contrast, *Dmrt1^−/−^* XY animals had severely reduced serum and gonadal testosterone levels, consistent with their hypovirilization (Fig. 8a and b). Serum FSH and LH were both severely reduced in *Dmrt1^−/−^* XY animals, possibly as a result of highly elevated inhibin B (Fig. 8e). The low LH level in null mutant XY animals would be expected to reduce androgen production in Leydig cells and may help explain the reduced testosterone levels we observed. Hormonal signaling also was significantly disrupted in *Dmrt1^R111G^*^/+^ XY animals, but differently from that of null mutants. Serum and gonadal testosterone levels were normal, as was serum LH, indicating normal Leydig cell function (Fig. 8a–c). However, FSH was elevated more than 2-fold, possibly due to a 2-fold reduction in inhibin B (Fig. 8d and e). In summary, both the *Dmrt1* null allele and the R111G mutation disrupt HPG signaling but R111G has a strong dominant phenotype that affects Sertoli cell inhibin B signaling and FSH levels in the opposite direction to that of the null mutation.

### *Dmrt1^R111G^* causes dominant transcriptome feminization

**Fig. 9.**
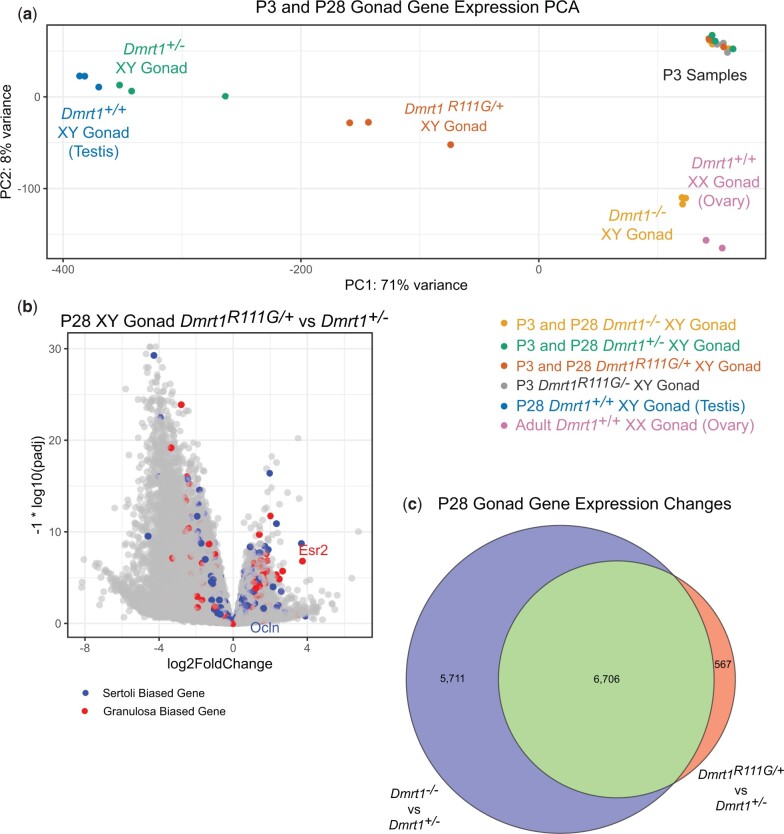
Dominant effect of *Dmrt1^R111G/+^* on gene expression. a) PCA analysis of P3 and P28 gonads. b) Volcano plot of mRNA expression changes in P28 *Dmrt1^R111G/+^* XY gonads relative to *Dmrt1^+/−^* XY control gonads. c) Venn diagram of mRNA expression changes in *Dmrt1^+/−^* vs *Dmrt1^−/−^* XY gonads and *Dmrt1^R111G/+^* vs *Dmrt1^+/−^* XY gonads.

The dominant testis phenotype of the *Dmrt1^R111G^* allele and the dominant effect of DMRT1^R111G^ protein on DNA binding in vitro (Murphy *et al.* 2015) both suggest that *R111G* is a neomorphic allele rather than a strict loss-of-function allele. To further investigate this possibility we first performed mRNA profiling, asking whether *Dmrt1^R111G^*^/+^ affects the transcriptome similarly to *Dmrt1* loss of function or has distinct characteristics. To find any very early changes we examined P3 as well as P28 (Supplementary Table 1). At P3, wild-type, *Dmrt1^-/+^*, *Dmrt1^−/−^*, and *Dmrt1^R111G/+^* XY gonads clustered tightly together in PCA, indicating only minor differences in gene expression (Fig. 9a). At P28, however, major changes were evident. Wild-type P28 XX ovaries were located close to P3 XY gonads in PC1, which accounted for 71% of variance, but separated in PC2, which accounted for 8% of variance (Fig. 9a). Wild-type and *Dmrt1^-/+^* P28 XY gonads were widely separated from wild-type ovaries in both PC1 and PC2, while *Dmrt1^−/−^* P28 XY gonads clustered close to wild-type ovaries. Strikingly, *Dmrt1^R111G/+^* P28 XY gonads were intermediate between wild-type testes and ovaries in both PC1 and PC2. We identified 6,032 mRNAs that were expressed more highly in *Dmrt1^+/−^* XY gonads and 1,241 mRNAs expressed more highly in *Dmrt1^R111G/+^* XY gonads with a >2-fold change and a Benjamini–Hochberg adjusted *P*-value below 0.05, but notable neither Sox9 nor Foxl2 were differentially expressed between these 2 genotypes (Supplementary Table 1). Thus, although *Dmrt1^R111G/+^* XY gonads retained SOX9 expression and did not express high levels of *Foxl2*, the mutation caused dominant partial feminization of the transcriptome. The dominant partial feminization of *Dmrt1^R111G/+^* P28 XY gonads also was apparent in a volcano plot (Fig. 9b). Known Sertoli- and granulosa-biased mRNAs were misexpressed, but the pattern was more mixed than in null mutant gonads, with many members of both gene classes upregulated and downregulated. Nevertheless, more than half of the mRNAs that were misregulated in null mutant gonads also were affected in *Dmrt1^R111G/+^* P28 XY gonads (Fig. 9c). Additionally, expression of 567 mRNAs was affected in *Dmrt1^R111G/+^* but not in *Dmrt1^−/−^* or *Dmrt1^+/−^* mutants (Fig. 9c), suggesting that this allele has neomorphic effects on gene expression.

### R111G is a neomorphic allele of *Dmrt1* that alters DNA binding in vivo

**Fig. 10.**
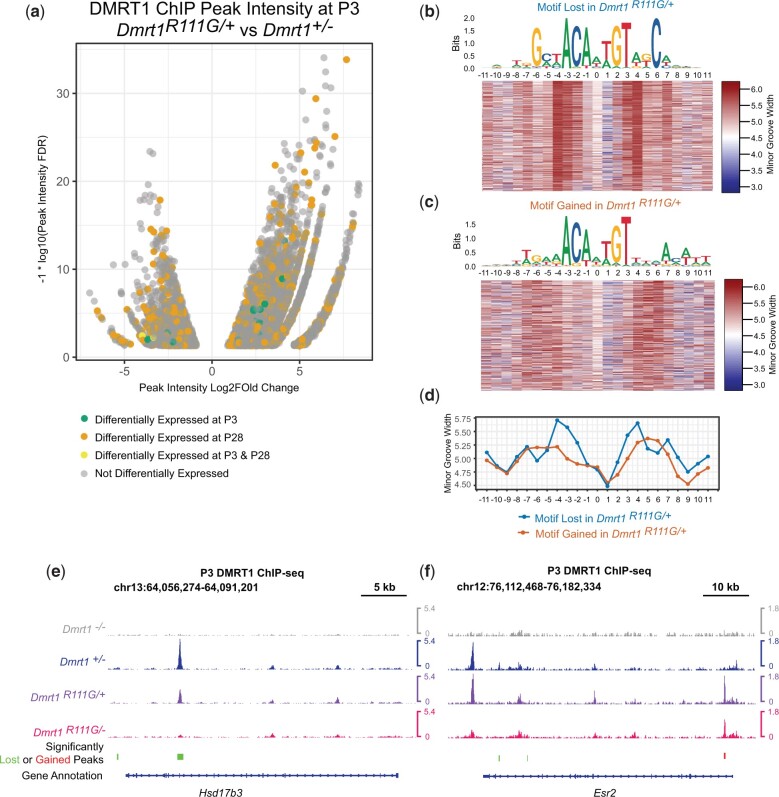
Altered DNA binding by DMRT1 in *Dmrt1^R111G/+^* XY gonads. a) Volcano plot showing sites differentially bound by DMRT1 in *Dmrt1^R111G/+^* XY gonads relative to *Dmrt1^+/−^* XY control testes. Sites associated with genes differentially expressed at P3 and/or P28 are shaded. b) Motif enriched in sites showing reduced binding by DMRT1 in *Dmrt1^R111G/+^* XY gonads and heatmap of predicted minor groove width across bound peaks. c) Motif enriched in sites showing increased binding by DMRT1 in *Dmrt1^R111G/+^* XY gonads and heatmap of predicted minor groove width across bound peaks. d) Plot of predicted minor groove width across motifs gained and lost in *Dmrt1^R111G/+^* XY gonads. Binding by DMRT1 to transcribed regions of *Hsd17b3* (e) and *Esr2* (f), showing altered affinity of DMRT1 in *Dmrt1^R111G/+^* XY gonads relative to wild-type XY testes and *Dmrt1^+/−^* XY control testes. Regions with reduced binding in *Dmrt1^R111G/+^* XY gonads are indicated by green bars and gained binding by red bars below the tracks.

Next, we used ChIP-seq to examine the distribution of DMRT1 DNA binding, comparing gonads of *Dmrt1^R111G^*^/+^ P3 males to those of P3 heterozygotes (*Dmrt1^+/−^*) in order to find early anomalies that might help trigger transdifferentiation. Previous in vitro studies indicated that the R111G allele allows the mutant DMRT1 protein to alter binding by wild-type DMRT1 (Murphy *et al.* 2015), and we asked whether this also is true in vivo. We identified 9,311 peaks with increased or novel binding in *Dmrt1^R111G^*^/+^ testes and 2,697 peaks with decreased intensity. The binding by DMRT1 to inappropriate sites confirms that the R111G mutation alters DNA binding specificity in vivo and causes aberrant DMRT1 DNA association prior to extensive effects on gene expression. Altered DNA binding causes altered gene expression: among the affected sites are some associated with genes differentially expressed at P3, P28, or both in *Dmrt1^R111G^*^/+^ testes (Fig. 10a). We searched for genes associated with the affected peaks that have Sertoli- or granulosa-biased expression or known roles in sex determination, and identified 40 such genes, associated with 82 differentially bound peaks. These genes include sexual regulators like *Esr2* and the androgenic enzyme *Hsd17b3*, which may contribute to the observed phenotype.

To learn more about how R111G affects DMRT1 DNA binding in vivo, we examined the sites that showed increased or reduced binding in *Dmrt1^R111G^*^/+^ testes, deriving consensus DNA sequences and examining predicted DNA shape (Fig. 10b and c). As mentioned earlier, DMRT1 can bind DNA as a dimer, trimer, or tetramer in vitro, and each stoichiometry is associated with a different DNA sequence and shape (Murphy *et al.* 2015). The sites showing reduced binding in *Dmrt1^R111G^*^/+^ had the typical sequence motif associated with tetramer binding and also had the predicted bilateral wide minor groove at positions −4 and +4 (Fig. 10b and d). The sites showing enhanced binding in *Dmrt1^R111G^*^/+^ lacked the bilateral wide minor groove and the G/C bases at −6/+6 (Fig. 10c and d), suggesting they were not tetramer sites, but might be dimer sites. They also had a nascent ACA sequence motif on the right side that could allow a novel mode binding by a third DMRT1 protomer. Two examples of changes in DNA binding are shown. At *Hsd17b3*, R111G, either heterozygous or hemizygous, resulted in weaker binding to a site normally bound by DMRT1, with the hemizygote more severely affected (Fig. 10e). At *Esr2*, *Dmrt1^R111G^*^/+^ mutants had strong binding to the sites bound in normal control testes, but also a novel binding site that also was present in *Dmrt1^R111G^*^/^^*−*^ mutants (Fig. 10f). Together these ChIP results further support the idea that R111G is a neomorphic allele that dominantly alters DNA-binding specificity and affects expression of many DMRT1 target genes as well as other genes not normally regulated by DMRT1.

### R111G does not activate ESR2 and LRH1 activity

**Fig. 11.**
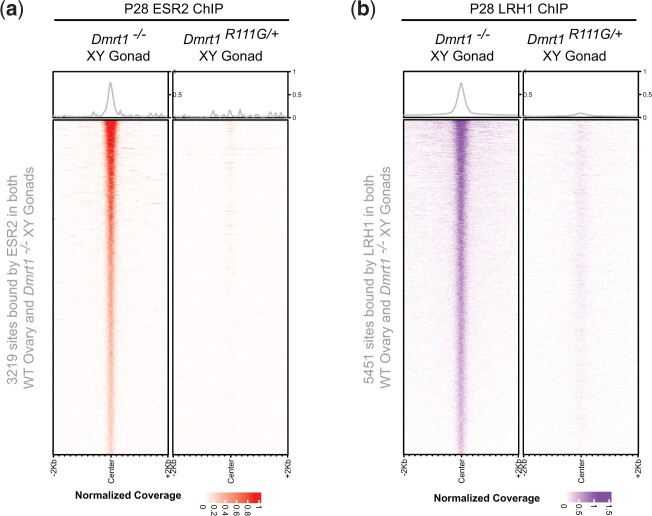
R111G mutation does not activate DNA binding by ESR2 or LRH1. a) Tornado plot showing binding by LRH1 in *Dmrt1^R111G/+^* XY gonads to sites bound by LRH1 both in wild-type ovaries and in *Dmrt1^−/−^* XY gonads. Left plot shows binding in *Dmrt1^−/−^* XY gonads and right plot shows binding in *Dmrt1^R111G/+^* XY gonads. b) Tornado plot showing binding by ESR2 in *Dmrt1^R111G/+^* XY gonads to sites bound by ESR2 in wild-type ovaries and *Dmrt1^−/−^* XY gonads. Left plot shows binding in *Dmrt1^−/−^* XY gonads and right plot shows binding in *Dmrt1^R111G/+^* XY gonads.

As discussed above in *Dmrt1* null mutant XY gonads, ESR2 and LRH1 are expressed at elevated levels and function to drive transdifferentiation. We therefore asked whether these 2 female regulators are active in *Dmrt1^R111G^*^/+^ XY gonads. We performed ChIP-seq for ESR2 and LRH1 and examined binding to the sites that were bound by these proteins in both ovaries and *Dmrt1^−/−^* XY gonads (Fig. 11). Although *Esr2* expression was elevated at P28, ESR2 did not bind DNA in the *Dmrt1^R111G^*^/+^ XY mutant gonads at sites previously shown to be bound by ESR2 in both wild-type XX ovaries and *Dmrt1^−/−^* XY gonads. ESR2 requires an estrogen ligand for nuclear localization and DNA binding, so this result suggests that estrogen signaling is not activated in *Dmrt1^R111G^*^/+^ mutants. Consistent with this possibility, the estrogenic enzyme *Cyp19a1* was strongly downregulated in *Dmrt1^R111G^*^/+^ mutant gonads (Supplementary Table 1). *Lrh1* expression was not elevated in *Dmrt1^R111G^*^/+^ XY gonads (Supplementary Table 1) and as expected there was no significant binding in these mutants compared with *Dmrt1^−/−^* XY gonads.

## Discussion

Here, we have investigated how DMRT1 maintains male sexual differentiation in testicular Sertoli cells and how the human sex-reversing *DMRT1^R111G^* mutation alters DMRT1 function. We confirmed by RNA-seq that complete loss of *Dmrt1* function causes a postnatal transformation of the testis transcriptome to a transcriptional profile resembling that of the ovary. This transformation begins by P7, prior to the overt differences in morphology and cell lineage marker expression we previously documented, and it involves inappropriate conversion between active and inactive chromatin compartments at likely sex-regulatory genes. We modeled *DMRT1^R111G^* in the mouse and found that it causes dominant testis differentiation defects and partially feminizes the XY gonadal transcriptome, consistent with a causative role in human gonadal dysgenesis. ChIP-seq and RNA-seq analysis indicated that *Dmrt1^R111G^* is a neomorphic allele rather than a strict loss of function, as the mutant protein interacts with chromatin aberrantly, including both failure to bind at some of its normal sites and binding to inappropriate sites, and it appears to regulate some genes not normally controlled by *Dmrt1*.

We asked whether female regulators that are activated by DMRT1 and drive feminization function in a manner similar to their roles in the ovary or via distinct regulatory networks. ChIP-seq showed that FOXL2, ESR2 and LRH1 are able to associate with chromatin in *Dmrt1* mutant XY gonads, and that they bind many of the same sites bound in granulosa cells of the wild-type ovary. This result suggests that the regulatory networks driving male-to-female transdifferentiation at least partially resemble those driving normal female differentiation. Additionally, the association of ESR2 with DNA indicates that estrogen signaling is sufficiently activated in the null mutant gonads to allow nuclear localization of ESR2. The overlap in binding profiles of the 2 nuclear receptor proteins ESR2 and LRH1 suggests possible cooperation or redundancy in regulation of target genes. Whether the 2 proteins can bind DNA sites simultaneously and whether they physically interact will require further study. The mix of targets bound individually and jointly by the 3 female sex regulators suggests that they collectively drive feminization by regulating distinct but overlapping suites of target genes.

We showed previously that ectopic DMRT1 in the ovary can act in the manner of a pioneer transcription factor, binding and opening inaccessible chromatin and allowing other transcription factors, for example SOX9, to bind and regulate transcription (Lindeman *et al.* 2021). Here, we have identified chromatin regions whose activity in juvenile Sertoli cells is dependent on DMRT1. These regions are good candidates to play a key role in the maintenance of male cell fate and the initiation of transdifferentiation. Some genes that later are highly overexpressed and are known to be important for transdifferentiation, for example, *Esr2* and *Lrh1*, were not activated at this early stage and did not show abnormal chromatin activity. This difference suggests that sexual transdifferentiation may occur in distinct phases, perhaps involving a reset of sexual cell identity followed by redifferentiation.

Human genetics has indicated that DMRT1 is required in 2 copies for testis differentiation. A variety of microdeletions and point mutations in DMRT1 have been identified in XY individuals with partial or complete gonadal dysgenesis, often leading to male-to-female phenotypic sex reversal (Zarkower and Murphy 2021). The evidence is therefore strong that mutations in DMRT1 are causative for human XY gonadal dysgenesis. It also is clear that genetic background affects sensitivity to *DMRT1* mutations, as deletions removing *DMRT1* vary in severity and siblings carrying the same mutation can have different degrees of gonadal dysgenesis (Calvari *et al.* 2000). Similarly, the requirement for DMRT1 differs between species: loss of just 1 copy of human *DMRT1* can cause complete gonadal dysgenesis and sex reversal, while in mice *Dmrt1* loss of function is recessive and even XY homozygous null mutants are born phenotypically male. We did, however, detect some abnormalities in testicular hormone signaling in heterozygous mutants, suggesting that even in the mouse *Dmrt1* activity may be weakly dose-dependent.

We modeled the human *DMRT1^R111G^* mutation in mouse for 2 reasons. First, we sought direct evidence that a point mutation can sufficiently compromise DMRT1 to cause severe gonadal defects. Second, we sought to test the prediction from human genetics and in vitro DNA-binding analysis that the mutation might have a dominant phenotype. The mouse model confirmed both predictions. *Dmrt1^R111G/+^* XY animals developed testes with both somatic and germ line defects, including a disrupted BTB and highly reduced spermatogonial differentiation, and the transcriptome was severely disrupted and partially feminized. In principal, these defects may stem from cell autonomous disruptions in both cell types. A second and nonexclusive possibility is that the germ cell defects result indirectly from defects in the Sertoli cells that affect their ability to support germ cell differentiation. In particular, the lack of BTB integrity that we observed could potentially explain much of the failure of spermatogonia in the mutant testes to undergo meiosis and postmeiotic differentiation, as these events require an intact BTB (Kaur *et al.* 2014).

The dominant basis of the *R111G* phenotype was evident in ChIP-seq analysis, as heterozygous mutant gonads had inappropriate localization of DMRT1, despite the presence of a wild-type *Dmrt1* allele. Three classes of target genes were noteworthy for their response to the *R111G* mutation. First, and most straightforward, many genes affected in null mutants were also affected in *Dmrt1^R111G/+^* XY animals and were misregulated similarly in both types of mutants. We found that the R111G mutation selectively reduced binding to tetramer consensus sites, so this first class of genes may be regulated via tetramer binding sites that lose binding in *Dmrt1^R111G/+^* XY animals. Second, some genes normally regulated by DMRT1 responded differently in *Dmrt1^R111G/+^* XY animals relative to null mutants. We showed previously that DMRT1^R111G^ can alter binding stoichiometry of wild-type DMRT1 (Murphy *et al.* 2015) and altered stoichiometry may explain many of the expression changes. Third, more than 500 genes were misregulated in *Dmrt1^R111G/+^* XY animals but not in null mutants. We observed a gain of affinity at potential dimer or trimer sites and this gain of binding likely contributes to misregulation of novel genes. Different alleles of DMRT1, including microdeletions and point mutations, have been found to cause XY gonadal dysgenesis and male-to-female sex reversal. Our comparison of a *Dmrt1* null mutation and the *Dmrt1^R111G/+^* point mutation suggests that strong human *DMRT1* loss-of-function alleles may primarily affect gonadal sex differentiation, while *DMRT1^R111G^* may cause a mix of gonadal feminization and more general developmental disruption unrelated to sexual cell fate. The DMRT1-dependent mRNAs and chromatin regions we have identified in mouse models may help to understand the etiology of the human phenotypes.

## Supplementary Material

jkac267_Supplementary_Data_Figure_S1

jkac267_Supplementary_Data_Figure_S2

jkac267_Supplementary_Data_Figure_S3

jkac267_Supplementary_Supplemental_Material

jkac267_Supplementary_Table_S1

jkac267_Supplementary_Table_S2

## Data Availability

Genomic data generated for this paper are available through the Gene Expression Omnibus with accession number GSE208106 and published data used in this paper are available with accession numbers GSE60858 (Georges *et al.* 2014) and GSE154484 (Lindeman *et al.* 2021). Supplemental material is available at G3 online.

## References

[jkac267-B1] Agrimson KS , Minkina A, Sadowski D, Wheeler A, Murphy MW, Gearhart MD, Bardwell VJ, Zarkower D. *Lrh1* can help reprogram sexual cell fate and is required for Sertoli cell development and spermatogenesis in the mouse testis. PLoS Genet. 2022;18(2):e1010088. 35192609 10.1371/journal.pgen.1010088 PMC8896720

[jkac267-B2] Albrecht KH , Eicher EM. Evidence that Sry is expressed in pre-Sertoli cells and Sertoli and granulosa cells have a common precursor. Dev Biol. 2001;240(1):92–107. 11784049 10.1006/dbio.2001.0438

[jkac267-B3] Arnold AP. Four core genotypes and XY mouse models: pdate on impact on SABV research. Neurosci Biobehav Rev. 2020;119:1–8. 32980399 10.1016/j.neubiorev.2020.09.021 PMC7736196

[jkac267-B4] Barrionuevo FJ , Hurtado A, Kim GJ, Real FM, Bakkali M, Kopp JL, Sander M, Scherer G, Burgos M, Jiménez R, et al *Sox9* and *Sox8* protect the adult testis from male-to-female genetic reprogramming and complete degeneration. Elife. 2016;5:e15635. 27328324 10.7554/eLife.15635 PMC4945155

[jkac267-B5] Buonocore F , Clifford-Mobley O, King TFJ, Striglioni N, Man E, Suntharalingham JP, Del Valle I, Lin L, Lagos CF, Rumsby G, et al Next-generation sequencing reveals novel genetic variants (*SRY*, *DMRT1*, *NR5A1*, *DHH*, *DHX37*) in Adults With 46,XY DSD. J Endocr Soc. 2019;3(12):2341–2360. 31745530 10.1210/js.2019-00306 PMC6855215

[jkac267-B6] Calvari V , Bertini V, De Grandi A, Peverali G, Zuffardi O, Ferguson-Smith M, Knudtzon J, Camerino G, Borsani G, Guioli S, et al A new submicroscopic deletion that refines the 9p region for sex reversal. Genomics. 2000;65(3):203–212. 10857744 10.1006/geno.2000.6160

[jkac267-B7] Chauhan V , Jyotsna VP, Jain V, Khadgawat R, Dada R. Novel heterozygous genetic variants in patients with 46,XY Gonadal dysgenesis. Horm Metab Res. 2017;49(1):36–42. 27711951 10.1055/s-0042-114778

[jkac267-B8] Chiu T-P , Comoglio F, Zhou T, Yang L, Paro R, Rohs R. DNAshapeR: an R/Bioconductor package for DNA shape prediction and feature encoding. Bioinformatics. 2016;32(8):1211–1213. 26668005 10.1093/bioinformatics/btv735 PMC4824130

[jkac267-B9] Couse JF , Hewitt SC, Bunch DO, Sar M, Walker VR, Davis BJ, Korach KS. Postnatal sex reversal of the ovaries in mice lacking estrogen receptors alpha and beta. Science. 1999;286(5448):2328–2331. 10600740 10.1126/science.286.5448.2328

[jkac267-B10] Duggavathi R , Volle DH, Mataki C, Antal MC, Messaddeq N, Auwerx J, Murphy BD, Schoonjans K. *Liver receptor homolog 1* is essential for ovulation. Genes Dev. 2008;22(14):1871–1876. 18628394 10.1101/gad.472008 PMC2492734

[jkac267-B11] Erdman SE , Burtis KC. The *Drosophila doublesex* proteins share a novel zinc finger related DNA binding domain. EMBO J. 1993;12(2):527–535. 8440242 10.1002/j.1460-2075.1993.tb05684.x PMC413235

[jkac267-B12] Fan Y , Zhang X, Wang L, Wang R, Huang Z, Sun Y, Yao R, Huang X, Ye J, Han L, et al Diagnostic application of targeted next-generation sequencing of 80 genes associated with disorders of sexual development. Sci Rep. 2017;7:44536. 28295047 10.1038/srep44536 PMC5353765

[jkac267-B13] Fayard E , Schoonjans K, Annicotte JS, Auwerx J. *Liver receptor homolog 1* controls the expression of carboxyl ester lipase. J Biol Chem. 2003;278(37):35725–35731. 12853459 10.1074/jbc.M302370200

[jkac267-B14] Gao P , Lyu Q, Ghanam AR, Lazzarotto CR, Newby GA, Zhang W, Choi M, Slivano OJ, Holden K, Walker JA, et al Prime editing in mice reveals the essentiality of a single base in driving tissue-specific gene expression. Genome Biol. 2021;22(1):83. 33722289 10.1186/s13059-021-02304-3 PMC7962346

[jkac267-B15] Garcia-Moreno SA , Futtner CR, Salamone IM, Gonen N, Lovell-Badge R, Maatouk DM. Gonadal supporting cells acquire sex-specific chromatin landscapes during mammalian sex determination. Dev Biol. 2019;446(2):168–179. 30594505 10.1016/j.ydbio.2018.12.023 PMC6368449

[jkac267-B16] Georg I , Barrionuevo F, Wiech T, Scherer G. *Sox9* and *Sox8* are required for basal lamina integrity of testis cords and for suppression of FOXL2 during embryonic testis development in mice. Biol Reprod. 2012;87(4):99. 22837482 10.1095/biolreprod.112.101907

[jkac267-B17] Georges A , L'Hote D, Todeschini AL, Auguste A, Legois B, et al The transcription factor FOXL2 mobilizes estrogen signaling to maintain the identity of ovarian granulosa cells. Elife. 2014;3:e04207. 25369636 10.7554/eLife.04207 PMC4356143

[jkac267-B18] Jauregui EJ , Mitchell D, Topping T, Hogarth CA, Griswold MD. Retinoic acid receptor signaling is necessary in steroidogenic cells for normal spermatogenesis and epididymal function. Development. 2018;145:dev160465. 29899137 10.1242/dev.160465 PMC6053667

[jkac267-B19] Jost A. Recherches sur la differenciation sexuelle del’embryon de lapin. III. Role des gonades foetales dans la differenciation sexuelle somatique. Arch Anat Microsc Morphol Exp. 1947;36:271–315.

[jkac267-B20] Kaur G , Thompson LA, Dufour JM. Sertoli cells-immunological sentinels of spermatogenesis. Semin Cell Dev Biol. 2014;30:36–44. 24603046 10.1016/j.semcdb.2014.02.011 PMC4043859

[jkac267-B21] Kim S , Bardwell VJ, Zarkower D. Cell type-autonomous and non-autonomous requirements for *Dmrt1* in postnatal testis differentiation. Dev Biol. 2007;307(2):314–327. 17540358 10.1016/j.ydbio.2007.04.046 PMC1995593

[jkac267-B22] Lenie S , Smitz J. Estrogen receptor subtypes localization shifts in cultured mouse ovarian follicles. Histochem Cell Biol. 2008;129(6):827–840. 18330591 10.1007/s00418-008-0408-9

[jkac267-B23] Lin YT , Capel B. Cell fate commitment during mammalian sex determination. Curr Opin Genet Dev. 2015;32:144–152. 25841206 10.1016/j.gde.2015.03.003 PMC4470863

[jkac267-B24] Lindeman RE , Gearhart MD, Minkina A, Krentz AD, Bardwell VJ, Zarkower D. Sexual cell-fate reprogramming in the ovary by DMRT1. Curr Biol. 2015;25:764–771. 25683803 10.1016/j.cub.2015.01.034 PMC4366330

[jkac267-B25] Lindeman RE , Murphy MW, Agrimson KS, Gewiss RL, Bardwell VJ, Gearhart MD, Zarkower D. The conserved sex regulator DMRT1 recruits SOX9 in sexual cell fate reprogramming. Nucleic Acids Res. 2021;49(11):6144–6164. 34096593 10.1093/nar/gkab448 PMC8216462

[jkac267-B26] Macheroni C , Lucas TFG, Porto CS. The role of estrogen receptors in rat Sertoli cells at different stages of development. Heliyon. 2020;6(11):e05363. 33163677 10.1016/j.heliyon.2020.e05363 PMC7609458

[jkac267-B27] Matson CK , Murphy MW, Sarver AL, Griswold MD, Bardwell VJ, Zarkower D. DMRT1 prevents female reprogramming in the postnatal mammalian testis. Nature. 2011;476(7358):101–104. 21775990 10.1038/nature10239 PMC3150961

[jkac267-B28] Meinsohn M-C , Hughes CHK, Estienne A, Saatcioglu HD, Pépin D, Duggavathi R, Murphy BD. A role for orphan nuclear receptor liver receptor homolog-1 (LRH-1, NR5A2) in primordial follicle activation. Sci Rep. 2021;11(1):1079. 33441767 10.1038/s41598-020-80178-4 PMC7807074

[jkac267-B29] Meinsohn M-C , Morin F, Bertolin K, Duggavathi R, Schoonjans K, Murphy BD. The orphan nuclear receptor *liver homolog receptor-1* (*Nr5a2*) regulates ovarian granulosa cell proliferation. J Endocr Soc. 2018;2(1):24–41. 29379893 10.1210/js.2017-00329 PMC5779114

[jkac267-B30] Minkina A , Matson CK, Lindeman RE, Ghyselinck NB, Bardwell VJ, Zarkower D. DMRT1 protects male gonadal cells from retinoid-dependent sexual transdifferentiation. Dev Cell. 2014;29(5):511–520. 24856513 10.1016/j.devcel.2014.04.017 PMC4105363

[jkac267-B31] Murphy MW , Lee JK, Rojo S, Gearhart MD, Kurahashi K, Banerjee S, Loeuille G-A, Bashamboo A, McElreavey K, Zarkower D, et al An ancient protein-DNA interaction underlying metazoan sex determination. Nat Struct Mol Biol. 2015;22(6):442–451. 26005864 10.1038/nsmb.3032 PMC4476070

[jkac267-B32] Murphy MW , Sarver AL, Rice D, Hatzi K, Ye K, Melnick A, Heckert LL, Zarkower D, Bardwell VJ. Genome-wide analysis of DNA binding and transcriptional regulation by the mammalian Doublesex homolog DMRT1 in the juvenile testis. Proc Natl Acad Sci USA. 2010;107(30):13360–13365. 20616082 10.1073/pnas.1006243107 PMC2922116

[jkac267-B33] Pérez CV , Sobarzo CM, Jacobo PV, Pellizzari EH, Cigorraga SB, Denduchis B, Lustig L. Loss of occludin expression and impairment of blood-testis barrier permeability in rats with autoimmune orchitis: effect of interleukin 6 on Sertoli cell tight junctions. Biol Reprod. 2012;87(5):122. 23018187 10.1095/biolreprod.112.101709

[jkac267-B34] Raymond CS , Murphy MW, O'Sullivan MG, Bardwell VJ, Zarkower D. *Dmrt1*, a gene related to worm and fly sexual regulators, is required for mammalian testis differentiation. Genes Dev. 2000;14(20):2587–2595. 11040213 10.1101/gad.834100 PMC316999

[jkac267-B35] Raymond CS , Shamu CE, Shen MM, Seifert KJ, Hirsch B, Hodgkin J, Zarkower D. Evidence for evolutionary conservation of sex-determining genes. Nature. 1998;391(6668):691–695. 9490411 10.1038/35618

[jkac267-B36] Schwabe JW , Chapman L, Finch JT, Rhodes D. The crystal structure of the estrogen receptor DNA-binding domain bound to DNA: how receptors discriminate between their response elements. Cell. 1993;75(3):567–578. 8221895 10.1016/0092-8674(93)90390-c

[jkac267-B37] Uda M , Ottolenghi C, Crisponi L, Garcia JE, Deiana M, Kimber W, Forabosco A, Cao A, Schlessinger D, Pilia G, et al *Foxl2* disruption causes mouse ovarian failure by pervasive blockage of follicle development. Hum Mol Genet. 2004;13(11):1171–1181. 15056605 10.1093/hmg/ddh124

[jkac267-B38] Uhlenhaut NH , Jakob S, Anlag K, Eisenberger T, Sekido R, Kress J, Treier A-C, Klugmann C, Klasen C, Holter NI, et al Somatic sex reprogramming of adult ovaries to testes by FOXL2 ablation. Cell. 2009;139(6):1130–1142. 20005806 10.1016/j.cell.2009.11.021

[jkac267-B39] Zarkower D , Murphy MW. DMRT1: an ancient sexual regulator required for human gonadogenesis. Sex Dev. 2021;1–14. 10.1159/000518272 PMC8885888 34515237

