# Supplementary figures and images for: Genomics of sexual cell fate transdifferentiation in the mouse gonad
Source: G3 (Bethesda). 2022 Oct 6;12(12):jkac267. doi: 10.1093/g3journal/jkac267 (PMC9713387; doi:10.1093/g3journal/jkac267)

A

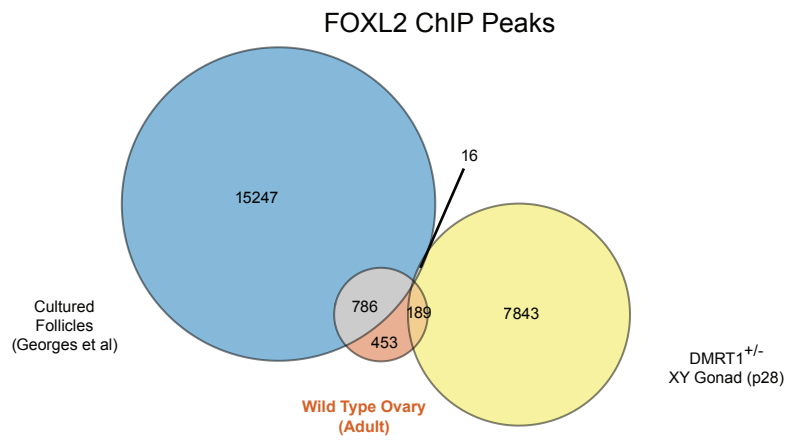

B

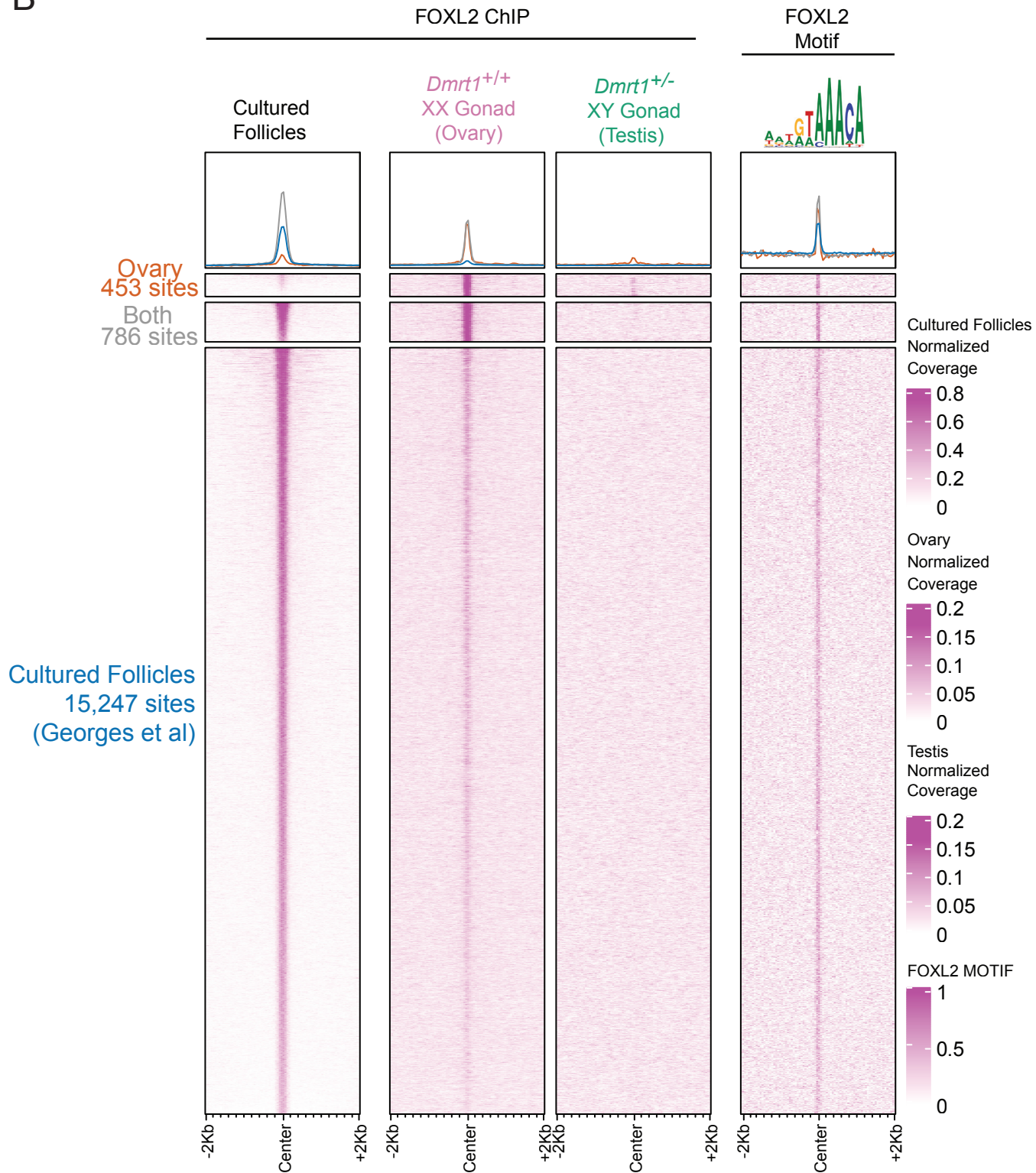

Supplement: jkac267_Supplementary_Data_Figure_S1 [file jkac267_supplementary_data_figure_s1.pdf]

# FOXL2 ChIP

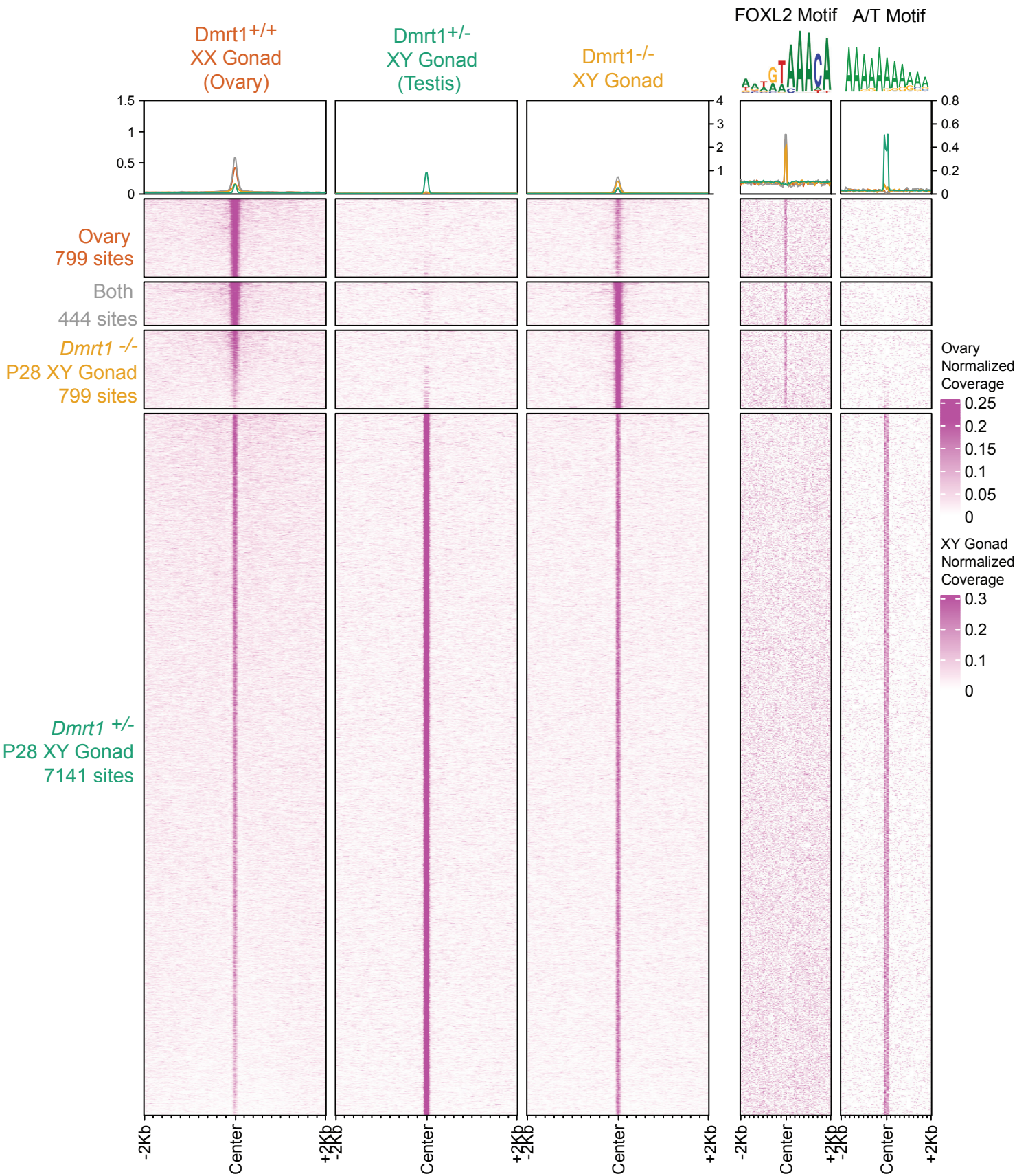

Supplement: jkac267_Supplementary_Data_Figure_S2 [file jkac267_supplementary_data_figure_s2.pdf]
