# Supplementary material for: Genomics of sexual cell fate transdifferentiation in the mouse gonad
Source: G3 (Bethesda). 2022 Oct 6;12(12):jkac267. doi: 10.1093/g3journal/jkac267 (PMC9713387; doi:10.1093/g3journal/jkac267)
Supplement: jkac267_Supplementary_Data_Figure_S3 [file jkac267_supplementary_data_figure_s3.pdf]

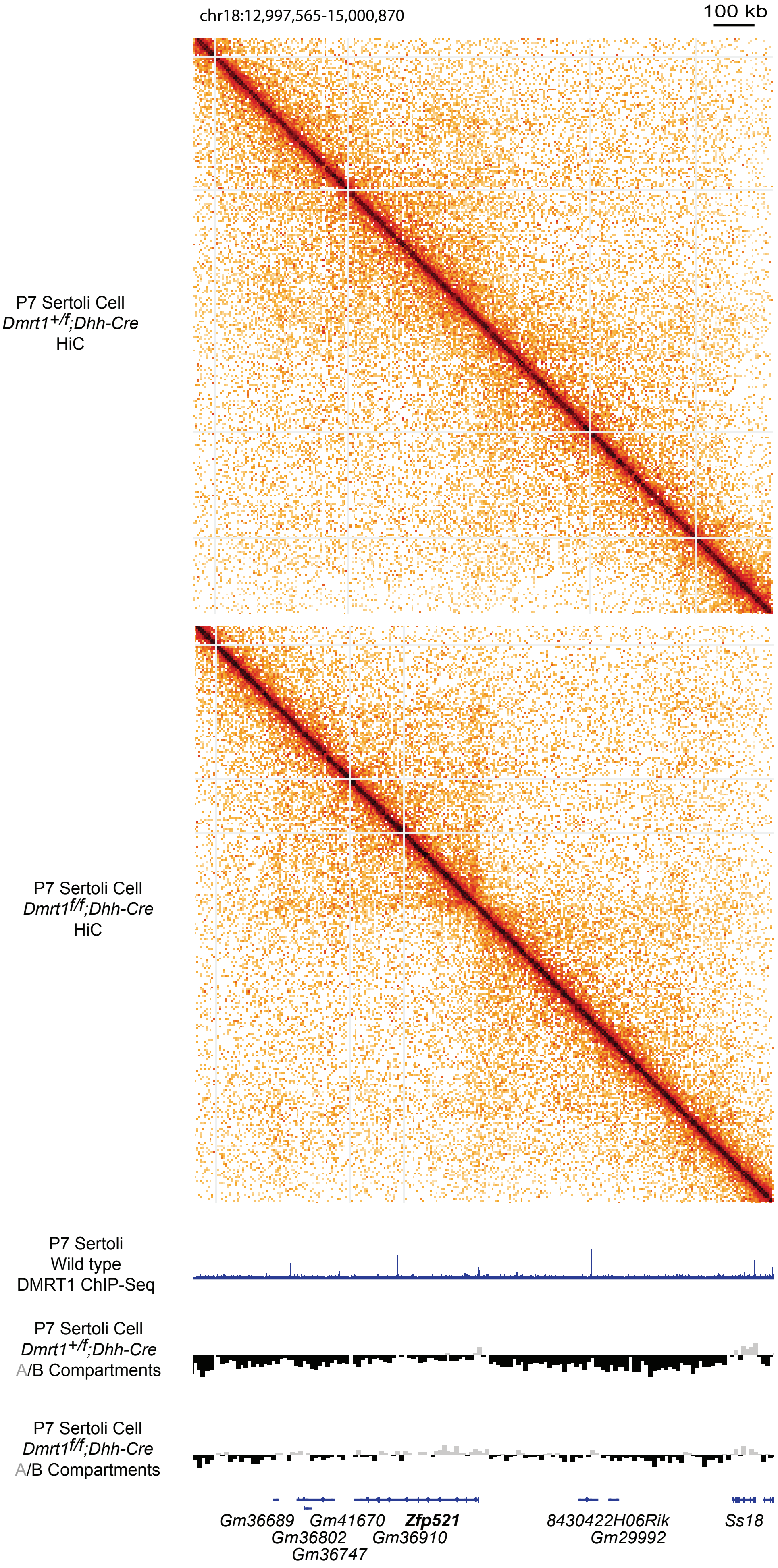
chr18:12,997,565-15,000,870
100 kb
P7 Sertoli Cell
Dmrt1+/f;Dhh-Cre
HiC
P7 Sertoli Cell
Dmrt1f/f;Dhh-Cre
HiC
P7 Sertoli
Wild type
DMRT1 ChIP-Seq
P7 Sertoli Cell
Dmrt1+/f;Dhh-Cre
A/B Compartments
P7 Sertoli Cell
Dmrt1f/f;Dhh-Cre
A/B Compartments
Gm36689
Gm41670
Zfp521
8430422H06Rik
Ss18
Gm36802
Gm36910
Gm29992
Gm36747
