# Supplementary material for: Genomics of sexual cell fate transdifferentiation in the mouse gonad
Source: G3 (Bethesda). 2022 Oct 6;12(12):jkac267. doi: 10.1093/g3journal/jkac267 (PMC9713387; doi:10.1093/g3journal/jkac267)
Supplement: jkac267_Supplementary_Supplemental_Material [file jkac267_supplementary_supplemental_material.docx]

**SUPPLEMENTAL FIGURE LEGENDS**

**Fig. S1 Validation of FOXL2 antibody in intact ovary.** (**A**) Venn diagram showing overlap in binding comparing ChIP-seq data from GEORGES *et al.* (2014) using cultured primary follicles with data from this study using whole ovaries or heterozygous control testes. (**B**) Tornado plot showing similar binding in cultured follicles (GEORGES *et al.* 2014) and wild type ovary (this study), and very low or absent binding in control testes, as well as canonical FOXL2 motif enriched in bound regions.

**Fig. S2 FOXL2 antibody detects distinct protein in testis.** Tornado plot showing sites primarily bound in ovary or mutant XY gonad or both, compared with control heterozygous XY testis. Sites bound in ovary, mutant XY gonad or both are enriched for FOXL2 binding consensus, while those bound most strongly in control testes are enriched for a string of A/T base pairs.

**Fig. S3 DMRT1 maintains genome organization at female-biased genes.** HiC contact maps (top) and one-dimensional tracks for two regions surrounding the female-biased gene *Zfp521*. Enriched off-diagonal contacts for wild type or mutant Sertoli cells using 10 kb binning of the contact maps are shown in top panels. One-dimensional representations of the enriched off-diagonal contacts, A/B compartments calculated from the eigenvalues of the interaction matrix, and DMRT1 ChIP-seq data as well as genomic features are shown in the tracks in the bottom panel.
